# The Nervous System Relevance of the Calcium Sensing Receptor in Health and Disease

**DOI:** 10.3390/molecules24142546

**Published:** 2019-07-12

**Authors:** Maria Lo Giudice, Balázs Mihalik, András Dinnyés, Julianna Kobolák

**Affiliations:** 1 BioTalentum Ltd., H-2100 Gödöllő, Hungary; 2 Molecular Animal Biotechnology Laboratory, Szent István University, H-2100 Gödöllő, Hungary

**Keywords:** calcium-sensing receptor, nervous system, calcilytic, calcimimetic, ischemia, Alzheimer’s disease, neuroblastoma

## Abstract

The calcium sensing receptor (CaSR) was first identified in parathyroid glands, and its primary role in controlling systemic calcium homeostasis by the regulation of parathyroid hormone (PTH) secretion has been extensively described in literature. Additionally, the receptor has also been investigated in cells and tissues not directly involved in calcium homeostasis, e.g., the nervous system (NS), where it plays crucial roles in early neural development for the differentiation of neurons and glial cells, as well as in the adult nervous system for synaptic transmission and plasticity. Advances in the knowledge of the CaSR’s function in such physiological processes have encouraged researchers to further broaden the receptor’s investigation in the neuro-pathological conditions of the NS. Interestingly, pre-clinical data suggest that receptor inhibition by calcilytics might be effective in counteracting the pathomechanism underlying Alzheimer’s disease and ischemia, while a CaSR positive modulation with calcimimetics has been proposed as a potential approach for treating neuroblastoma. Importantly, such promising findings led to the repurposing of CaSR modulators as novel pharmacological alternatives for these disorders. Therefore, the aim of this review article is to critically appraise evidence which, so far, has been yielded from the investigation of the role of the CaSR in physiology of the nervous system and to focus on the most recent emerging concepts which have reported the receptor as a therapeutic target for neurodegeneration and neuroblastic tumors.

## 1. Introduction

The calcium sensing receptor (CaSR) is a member of family C G-protein coupled receptors (GPCRs), and it was first cloned from bovine parathyroid gland in 1993 [1]. The human CaSR gene maps on chromosome 3q, and it encodes for a 1078 amino acids (aa) protein [2,3]. The CaSR protein presents the classic family C type GPCR structure with a very large extracellular N-terminal domain (ECD), a seven transmembrane domain (7TM) and an intracellular C-terminal domain (ICD) [1]. The CaSR-ECD, whose crystal structure has been recently reported [4,5], contains a venus flytrap (VFT) domain, through which the receptor binds its physiological ligand, the calcium ion (Ca^2+^), as well as various other agonists [6]. The VFT domain is linked via a cysteine-rich region to the 7TM, composed of seven α-helices (TM1–TM7) joined together by extracellular and intracellular loops. The 7TM region presents additive binding sites for Ca^2+^ and allosteric modulators [7,8]. The final TM α-helix is connected to the ICD, which is especially crucial for mediating the intracellular signaling [9] and for the negative regulation of the receptor [10,11].

In line with other GPCRs, the CaSR mediates a biased-signaling that is the capacity of the receptor to respond to a number of agonists that activate distinct intracellular signaling cascades in a tissue-dependent manner [12,13,14]. Indeed, the CaSR is sensitive to several different orthosteric agonists other than Ca^2+^, including the polyvalent cations Mg^2+^, Al^3+^, Sr^2+^, Mn^2+^, Gd^3+^ and some antibiotics such as neomycin, gentamycin and tobramycin [15]. Moreover, the polyamines spermine and spermidine, as well as the amyloid β peptide, were also reported to be involved in CaSR activation [16]. In contrast to the orthosteric agonists, which are able to activate the receptor on their own, the allosteric modulators are capable of modifying the receptor’s mediated signaling in the presence of an orthosteric ligand, e.g., the amino acids L-phenylalanine and L-tryptophan, both of which enhance receptor sensitivity to extracellular Ca^2+^ [17]. Synthetic positive and negative allosteric modulators, named calcimimetics and calcilytics, respectively, have been further developed in order to treat disorders of Ca^2+^ metabolism [18]. Such compounds can potentiate or decrease CaSR activated signaling. Evidences collected so far demonstrate that the CaSR can signal through distinct G protein-coupled pathways. These include the activation of G-protein Gq/11, which stimulates phospholipase C (PLC) to form inositol 1,4,5 tris-phosphate (IP_3_) and diacyl glycerol (DAG) [19]. As a consequence, IP_3_ promotes intracellular Ca^2+^ mobilizations, while DAG activates protein kinase C (PKC) and mitogen-activated protein kinase (MAPK) cascade [20]. Moreover, the CaSR may also promote the G_i/o_ pathway, which inhibits cyclic adenosine monophosphate (cAMP) production [21], and the G_12/13_ which, in turn, stimulates the rho kinase signaling [22]. In addition, the CaSR can mediate the recruitment of β-arrestins, which induce ERK1/2 phosphorylation [21] and regulate CaSR desensitization [23].

Being mainly expressed in the chief cells of the parathyroid glands, the CaSR exerts its primary role in sensing changes in extracellular Ca^2+^ and in maintaining Ca^2+^ homeostasis by regulating the secretion of PTH [24,25]. Indeed, when plasma Ca^2+^ increases, the activated CaSR inhibits PTH secretion. Conversely, in the case of hypocalcemia, the receptor is inactive, and the consequent release of parathyroid hormone restores Ca^2+^ concentration within the physiological range by promoting Ca^2+^ resorption from kidney and bone and Ca^2+^ absorption in the intestine [26]. To negatively regulate PTH secretion from parathyroid cells, the CaSR operates through the Gi/o-mediated inhibition of adenylate cyclase, which suppresses cAMP intracellular synthesis and the consequent release of parathyroid hormone [27,28]. Moreover, the activation of Gq/11-PLC signaling, associated with intracellular calcium Ca^2+^ mobilization, has been identified in parathyroid cells [29], and this mechanism has been hypothesized to contribute to the inhibition of adenylate cyclase [26]. Along with the role exerted in parathyroid glands, a growing body of evidence has demonstrated that the CaSR plays important and different functions in other tissues and organs. In the kidney, the CaSR is expressed in all nephron segments, and it has a role in the control of divalent mineral cation homeostasis, water and sodium reabsorption, and renin release [30], whereas in the stomach’s parietal cells, the receptor modulates gastric acid secretion via the regulation of the H+, K+ -ATPase [31]. Moreover, several reports demonstrated that the CaSR is also involved in the differentiation of adipocytes [32] and keratinocytes [33]. Additionally, the receptor regulates the chemotaxis of several cell types. Indeed, studies suggest that the CaSR supports the chemotaxis of osteoblast precursors to the site of resorption via calcium gradients [34], while it mediates the migration of immune cells through the PLC pathway [35]. Such evidences are in line with the CaSR biased signaling and strongly highlight the diversity of the receptor-mediated processes. Interestingly, a multiplicity of CaSR-mediated functions has also been observed in the nervous system, according to the cell type, developmental stage, and physiological condition of the NS. The first report of CaSR expression in the brain was provided in 1995, when Ruat and colleagues cloned the receptor from a rat striatal cDNA library and showed its presence in several brain areas such as the hypothalamus, striatum, hippocampus, cortex, cerebellum, and brainstem [36]. Afterwards, in vitro and in vivo studies revealed that the CaSR plays a crucial function in several mechanisms, from differentiation and migration to excitability and pathological processes. Considering that calcium homeostasis is a key player for neurodevelopment, neurotransmission, and neurodegeneration, and that its crucial role in such processes has been the focus of intense investigations [37,38,39], this review paper is intended to concentrate on the exquisite hypothesized functions of the CaSR in the NS and to (i) present the roles of the CaSR in the differentiating cells of the developing nervous system established so far; (ii) report the current state of knowledge on the receptor functions in the adult nervous system, especially regarding neurotransmission and synaptic plasticity; (iii) describe the potential use of CaSR-based therapeutics in disorders such as ischemia, Alzheimer’s disease and neuroblastoma; and (iv) propose alternative strategies to further investigate critical and unsolved issues regarding the receptor’s function and signaling in the nervous system.

## 2. Role of the CaSR for the Developing NS: Differentiation of Neural Cells

Calcium signaling is extremely important for the development of the nervous system. In vertebrates, Ca^2+^ signals are crucial regulators of the specification of neural versus epidermal ectoderm. Studies in *Xenopus laevis* embryos have demonstrated that spontaneous elevations of intracellular Ca^2+^ are restricted to the dorsal ectoderm cells, which originate the neural progenitors and never occur in ventral ectoderm cells, which give rise to the epidermis [40,41]. In addition to neural induction, calcium signaling also participates in the proliferation and differentiation of neurons and in the neuro-glia switch [42].

Consistent with the Ca^2+^ relevance for the NS, an involvement of the CaSR in the development of the nervous system appeared to be likely, and several in vitro and in vivo approaches have been used to test this hypothesis. At this point, an important premise regarding CaSR in vivo models is necessary. The murine knock-out (KO) model of the CaSR (CaSR-/-), which is a model of human neonatal severe hyperparathyroidism [43], displays serious hypercalcemia, hypophosphatemia, and increased serum PTH that contribute to bone abnormalities, delayed growth, and, finally, to premature death. Therefore, such a severe phenotype makes it difficult to distinguish between the direct effects of CaSR deficiency and the secondary effects of hyperparathyroidism. 

However, such lethal effects can be normalized by the constitutive genetic ablation of PTH. Indeed, mice with the CaR-/-/PTH-/- genotype survive to adulthood with no obvious difference in size or appearance relative to controls [44,45] allowing for the investigation of the impact of the CaSR in physiology. Liu and co-workers conducted studies in this regard. The authors compared the brain phenotypes of CaSR-/- mice, CaSR-/-/PTH-/- mice, and wild type (WT) animals [46]. Results indicated that post-natal day seven and 14 (P7 and P14, respectively) CaSR deficient mice had a reduced brain weight and size as well as a decreased expression of neuronal (RBFOX3, RNA Binding Fox-1 Homolog 3, formerly NEUN) and glial (GFAP, Glial Fibrillary Acidic Protein; and MBP, Myelin Basic Protein) differentiation markers compared with age-matched WT animals [46]. Interestingly, CaSR-/-/PTH-/- mice showed a normal brain size and weight, thus indicating that the reduced brain growth of the CaSR-null mice was likely an indirect consequence of the receptor deficiency. However, the CaSR-/-/PTH-/- animals failed to completely escape the brain differentiation defects, suggesting a direct role of the CaSR in brain cell maturation. In agreement with evidence from in vivo models, in vitro analyses of neural stem cells (NSCs) derived from the subventricular zone (SVZ) of new-born CaSR-/- mice exhibited a delayed ability to differentiate, despite maintaining a normal proliferation capacity, compared with wild-type controls [46]. Overall, this report shows an involvement of the CaSR in brain differentiation. Many studies have aimed to better elucidate the role of the receptor in specific subtypes of differentiating neural cells, as described in the next sub-paragraphs and summarized in Table 1.

### 2.1. CaSR in Neuronal Differentiation

The role of the CaSR in regulating the axonal and dendritic growth of neurons in the peripheral and central nervous system has been extensively studied. In sympathetic neurons isolated from the mice superior cervical ganglion (SCG), CaSR mRNA reaches a peak of expression at embryonic day 18 (E18), which coincides with the time when the axons grow to innervate their targets [47]. By using several experimental approaches, including pharmacological modulation and receptor silencing, Vizard and co-workers demonstrated that activating the CaSR with high extracellular Ca^2+^ or with the calcimimetic NPS R-467 significantly increased axonal growth [48]. On the other hand, negative modulation, receptor deletion, or the expression of a dominant negative CaSR (DNCaSR) completely inhibited the effect of high Ca^2+^ on neurite outgrowth. A similar effect of the CaSR was also observed in dendritic extensions, as hippocampal neurons from DNCaSR mouse at post-natal day four (P4) developed significantly shorter dendrites than cells from wild-type animals [48]. More recently, Vizard et al. provided new insights in the field by presenting the key role of ERK1/2 signaling in CaSR-mediated neurite growth [49]. In particular, SCG-neurons overexpressing the receptor presented an increased phosphorylation of ERK1 and ERK2 upon stimulation with a high Ca^2+^. Pharmacological inhibition of ERK1/2 phosphorylation with U0126 efficiently blocked the Ca^2+^ mediated neurite growth, demonstrating that the CaSR mediated axonal growth depends on the ERK1/2 activated pathway [49]. 

Similar to what has been mentioned for osteoblasts and immune cells, in vitro and in vivo reports have suggested the involvement of the CaSR in neuronal migration and chemotaxis. Two gonadotropin-releasing hormone (GnRH) neuronal cell lines, GN11 and GT1-7, showed an increased chemotaxis when stimulated with high extracellular Ca^2+^, while such effect was attenuated when cells were transfected with DNCaSR [50]. Moreover, in GT1-7 cells, the stimulation of the CaSR by high Ca^2+^ and spermine led to the secretion of the monocyte chemoattractant protein-1 (MCP-1), which is known to support migration of rat neural stem cells [51]. Conversely, the expression of a DNCaSR significantly reduced the chemokine’s production induced by Ca^2+^ [50]. Additionally, CaSR-/-/PTH-/- mice presented a 27% reduction of GnRH neurons in the preoptic area (POA) of the anterior hypothalamus compared to the wild-type, revealing that a functional CaSR is required not only for the migration but also for the survival of the GnRH neuronal population [50].

Another example of CaSR involvement in neuronal chemotaxis and migration was recently reported in the developing cerebellum [52]. Cerebellar granule-cell precursor (GCPs) neurons are known to originate in the rhombic-lip and migrate rostrally to the external granule-cell layer (EGL) during development. There, GCPs proliferate, differentiate, and then exit the cell-cycle to undergo tangential migration within the EGL, followed by a radial migration to reach their final position in the internal granule cell layer (IGL) [52]. The study showed that, in the rat cerebellum, CaSR protein expression resulted to be strongly up-regulated from P7 to P18, a period which coincides with peak GCP migration. The authors demonstrated that the stimulation of the CaSR with calcimimetics NPS R-568 and NPS R-467 potentiated the laminin-mediated GCP migration, whereas the calcilytic NPS 2143 completely abolished this effect in vitro. Similarly to previous findings [49], the MAPK signaling was implicated, as GCP migration was found to be mediated by the phosphorylation of AKT and ERK2 in purified GCPs [52]. To further confirm their results, the authors injected DMSO, NPS R-467, or NPS 2143 into the cerebrospinal fluid of nine-days-old post-natal rats. By labeling pre-migratory dividing GCPs of the animals with BrdU, the granule cell migration was tracked from the EGL into the IGL over a span of 72 h. Interestingly, NPS 2143 increased the number of BrdU-positive GCPs in the EGL. Conversely, NPS R-467 reduced the number of BrdU-labeled GCPs in the EGL while enhancing the cells in the IGL compared to the DMSO control. These observations provided in vivo evidence that CaSR activation promotes radial migration from the EGL into the IGL [52]. Such studies support a role of the CaSR and MAPK signaling in the neuronal differentiation of the rodent brain. Whether the receptor plays a similar role in human brain development remains to be clarified.

### 2.2. CaSR Relevance in Oligodendrocyte Differentiation

Along with the described function in the neurons, several studies report the CaSR being involved in the maturation of oligodendrocytes. A CaSR genetic mutation, causing neonatal severe hyperparathyroidism (NSHTP), was associated with a paucity of deep white matter and a delayed myelination in a six-month-old infant [53]. Though it is difficult to distinguish between the aforementioned secondary effects of hyperparathyroidism from those of the mutation, this report implies a function of the CaSR in oligodendrocytes development. 

Consistent with this idea, evidence in CaSR-null mice showed that these animals had reduced levels of MBP in the cerebellum during cerebellar development, compared to the wild-type animals [54]. In vitro studies further corroborated this hypothesis. The expression of CaSR mRNA was found to be higher in rodent neural stem cells (NCS) which undergo differentiation towards the oligodendrocyte lineage—than in cells differentiating in neurons or astrocytes. A similar pattern of receptor expression was observed in adult and fully differentiated neural cells, with oligodendrocytes having the highest expression compared to cells from neuronal or astrocytic lineages [54]. Moreover, stimulation with high Ca^2+^ and spermine promoted the proliferation and maturation of oligodendrocytes progenitor cells (OPCs), while such processes were significantly blunted by the expression of a dominant negative CaSR [54,55]. Interestingly, in primary oligodendrocytes isolated from neonatal rats, stimulation with high Ca^2+^ or neomycin increased the open state probability (P_o_) of an outward K^+^ channel, an effect likely mediated by the CaSR [55]. In addition, an intracellular Ca^2+^ mobilization and inositol phosphate accumulation were observed in GalC-positive oligodendrocytes isolated from 20 days postnatal rat brain upon stimulation with high Ca^2+^ and with the positive allosteric modulator NPS R-568, demonstrating a CaSR-mediated activation of the phospholipase C pathway, similar to that observed in other cell types [56]. 

### 2.3. CaSR in Astrocyte Differentiation

Whether the CaSR has a comparable function to that described in the proliferation and development for cells of astrocytic lineage is still unresolved. Few studies, aimed to address this issue, have been carried out in glial-derived tumors cell lines. In U373 astrocytoma, the positive stimulation of the CaSR with increasing Ca^2+^, Gd^3+^, and neomycin was found to increase cell proliferation, as assessed by H3-thymidine-incorporation, and to activate nonselective cation channels (NCCs) [57]. Furthermore, in the U87 glioma cell line, CaSR activation by high Ca^2+^ or by NPS R-467 was responsible to mediate the opening of an outward K^+^ channel [58]. However, considering the limited number of studies and the usage of poorly relevant in vitro models, further investigations are required to determine the role of the CaSR in astrocyte development.

## 3. Role of CaSR for the Adult NS

### 3.1. Neurotransmission and Excitability

The maintenance and regulation of Ca^2+^ homeostasis is a crucial process for any cell, especially for such excitable cells as the neurons. The most important processes occurring in the nervous system, such as neurotransmission, synaptic plasticity, and excitability, depend on highly sophisticated machinery that finely control the intra- and extra-cellular Ca^2+^ level [59]. A role for the CaSR in regulating neuronal excitability and neurotransmission is supported by observations in humans. Subjects with autosomal dominant hypocalcemia (ADH), caused by activating mutations in the CaSR gene, present neonatal or childhood seizures [60]. Moreover, Kapoor and coworkers identified rare missense mutations in the CaSR genes of patients with idiopathic epilepsy [61]. A following study functionally characterized one of these variants, the R898Q, and found that this mutation increased the plasma membrane targeting of the mutated receptors, which is consistent with a gain-of-function phenotype [62]. However, subjects harboring this mutation unexpectedly displayed levels of serum calcium, phosphate, sodium, potassium, and parathyroid hormone within reference range, suggesting that the effect of the CaSR for epilepsy phenotypes is allele and tissue-specific [61].

Several in vitro and in vivo studies have attempted to investigate the function of the CaSR in neuronal calcium signaling and synaptic transmission. In this regard, few works indicated a role of the receptor in regulation of NCCs. The patch-clamp of rat hippocampal neurons demonstrated that the activation of the CaSR with high extracellular Ca^2+^, neomycin, or spermine significantly increased the opening state probability of nonselective cation channels in WT neurons but not in cells isolated from CaR-/- mice [63,64]. Similarly, the P_o_ of NCCs resulted to be enhanced by high Ca^2+^ stimulation in HEK-293 cells stably transfected with the CaSR but not in non-transfected cells, confirming a role of the receptor in the regulation of NCCs [65]. At the same time, another study reported that the CaSR functionally coupled to Ca^2+^-activated K^+^ channels (CAKCs), as stimulation with high extracellular Ca^2^ and neomycin increased the P_o_ of these channels in wild-type neurons but not in CaSR-deficient neurons. Authors hypothesized that the receptor’s mediated-increase of intracellular Ca^2+^ levels activated these K^+^ channels, leading to membrane hyperpolarization [66]. These findings supported the idea that the CaSR can detect changes in extracellular Ca^2^ and transduce them into changes in neuronal excitability. More recent reports have introduced new insights in this field [67,68,69]. In particular, patch-clamp recordings from isolated mice cortical terminals showed that reduction in extracellular Ca^2+^ at the synaptic cleft, as it occurs during synaptic activity, activated nonselective cation channels, while increases in an extracellular Ca^2+^ stimulated CaSR and reduced the channel currents [67,68]. In agreement, electrophysiological analyses on neurons from CaSR-/- mice exhibited increased excitatory post-synaptic currents (EPSC) compared to neurons from WT animals [68]. Afterwards, Lu and coworkers hypothesized that CaSR regulates an Na-leak channel non selective, NALCN [70]. Authors showed that, in rodent hippocampal neurons, reductions in extracellular Ca^2+^ activated an NALCN which mediated a depolarizing current and increased neuronal excitability. The activation of an NALCN occurred through a cascade which involved a Ca^2+^-sensing GPCR, presumably the CaSR, and two intracellular proteins, UNC-79 and UNC-80 [70,71]. Altogether, these studies support the idea that CaSR activation would depress neurotransmission, whereas reduced CaSR function would enhance synaptic transmission. Conversely, Vyleta and colleagues described the idea that the activation of the receptor mediates spontaneous glutamate release [72]. Indeed, by imaging intracellular Ca^2+^ in nerve terminals and by measuring miniature EPSCs (mEPSCs) in cultured mouse neocortical neurons, it was observed that the stimulation of the CaSR with calindol and cinacalcet (an allosteric activator of the CaSR which is approved for clinical use) increased spontaneous vesicle fusion and mEPSC frequency, with a consequent glutamate release. As a confirmation, the frequency of spontaneous synaptic transmission and glutamate secretion were decreased in neurons isolated from CaSR-/- mice [72]. Such controversial evidences would suggest that the activation of the CaSR might produce opposite effects on the evoked and spontaneous release of the major excitatory neurotransmitter. However, further studies are required to clarify the mechanism by which the receptor could control the release of these apparently different pools of glutamate [71]. In contrast with previous works, a recent report revealed that potent allosteric modulators of the CaSR had no effect on mEPSC frequency in adult mouse CA1 hippocampal pyramidal cells. Moreover, mRNA and protein analyses failed to detect any CaSR expression in adult hippocampal neurons [73]. To sum up, although some CaSR genetic mutations are associated with epilepsy and seizures in humans, which suggests a crucial role of the receptor in neuronal excitability, the lack of a comprehensive investigations together with the controversy—results in the limited reports on CaSR involvement in the physiology of mature neural cells, strongly highlight the need of further studies in order to make certain conclusions in this regard.

### 3.2. Heterodimerization with Other GPCRs

In addition to the CaSR, other GPCRs have been shown to sense and to be activated by extracellular Ca^2+^ ions; these include several metabotropic glutamate receptors (mGluRs) and γ-amino isobutyric acid B receptors (GABABRs) [74,75]. Indeed, it was reported that members of mGluRs are activated by Ca^2+^ at physiological concentrations and that a single amino acid residue is responsible to determine the sensitivity to extracellular Ca^2+^ [74]. Moreover, in vitro studies have demonstrated that Ca^2+^ acts allosterically to potentiate GABA responses at the GABAB receptors in membranes prepared from Chinese hamster ovary (CHO) cells stably expressing the GABABR1/R2 heterodimer as well as in membranes from rat brain cortex [75]. Similar to the CaSR, these “calcium sensors” belong to the family C of G-protein coupled receptors and share significant homology with the calcium-sensing receptor [1]. Furthermore, mGluRs and GABABRs represent critical receptors for neuronal activity. In consideration of the role of the CaSR in neurotransmission and excitability described above, it is noteworthy that the heterodimerization of the CaSR and GABABRs or mGluRs has been observed to occur in the brain. In 2001, Gama and collaborators reported the co-immunoprecipitation of the CaSR and mGluR1α from bovine brains, whereas the immunohistochemical co-localization of the receptors was showed in rat brain samples [76]. Such ability to heterodimerize was also confirmed in vitro. The receptors were found to form heterodimers in HEK-293 cells transiently transfected with both GPCRs, and the CaSR resulted to become sensitive to glutamate-mediated internalization when present in CaSR/mGluR1α heterodimers [76]. Similar in vitro and in vivo approaches were used to demonstrate heterodimerization between the CaSR and GABABRs. The co-immunoprecipitation of the CaSR and GABA type B receptor was reported from HEK-293 cells expressing these receptors and from mouse brain lysates [77]. Interestingly, authors suggested a regulatory effect of GABABR1 on CaSR levels. Indeed, the expression of the CaSR was increased in lysates from GABABR1 knock-out mouse brains and in cultured hippocampal neurons with their GABABR1 genes deleted in vitro [77]. By highlighting the importance of family C GPCRs dimerization, these studies added a further level of complexity in elucidating the role of the CaSR in the brain and raised questions regarding (i) how these interactions between GPCRs might affect ligand binding and sensitivity; (ii) what might be the biological meaning of such dimerization in the nervous system; (iii) how dimerization affects the pharmacology of the resulting receptor; and (iv) how dimerization modulates the signaling networks and neurotransmission. To date, these questions are yet to be answered.

## 4. CaSR as a Potential Target for Disorders of Nervous System

As above mentioned, the maintenance of Ca^2+^ homeostasis is extremely important for the nervous system, thus its dysfunction can seriously compromise the condition of NS cells. Importantly, disturbances in Ca^2+^ regulation and signaling can mediate different processes from brain ageing to pathological conditions according to the level of extent [78]. The physiological ageing of the brain is characterized by slow and subtle changes in Ca^2+^ equilibrium, which are mainly represented by increased intracellular Ca^2+^ transients as well as reduced Ca^2+^ buffering capacity, especially in mitochondrial compartments. Indeed, mitochondria contribute to this condition, with a decreased ability to sequester excess Ca^2+^ from cytosol and the augmented production of reactive oxygen species (ROS) in aged brains, as reviewed in the recent work of Muller and colleagues [79]. Together, such events lightly affect neurotransmission and synaptic plasticity, thus leading to a mild cognitive deficit. A different and more serious Ca^2+^ dysregulation is critically involved in the development of pathological conditions and neurodegenerative disorders. Remarkably, emerging evidences have reported the CaSR as having an impact in neurodegeneration and neuroblastic tumors and the receptor’s allosteric modulators as playing novel potential therapeutics. Such concepts will be widely described in the next sections.

### 4.1. Ischemia and Hypoxia

Ischemia is the interruption of blood flow to cells and tissues, causing decreased oxygen and nutrient consumption and the improper removal of ‘metabolic waste’ at the same time [80]. A consequence of ischemic brain injury is the aberrant activation of N-methyl-D-aspartate receptors (NMDARs) and α-amino-3-hydroxy-5-methyl-4-isoxazolepropionic acid receptors (AMPARs) due to an excessive release of the excitatory neurotransmitter glutamate [81]. This raises intracellular Ca^2+^, which promotes excitotoxicity and neuronal cell death [82]. 

Interestingly, it has been reported that CaSR expression is up-regulated in rat brains after transient focal cerebral ischemia [83]. In this study, CaSR expression was induced in both the ischemic and border zones but showed different patterns in these two regions. Six hours after reperfusion, the receptor expression in the ischemic zone was induced preferentially in neurons and cells associated with blood vessel as endothelial cells and pericytes. This effect was followed by a gradual and sustained induction of the CaSR in reactive astrocytes located in the border zone of the post-ischemic brain, three-to-fourteen days after reperfusion [83]. Furthermore, CaSR expression resulted to be upregulated in rat primary cortical astrocytes exposed to oxygen–glucose deprivation, which induces reactive gliosis-like changes of the cells [84]. These results strongly suggest an involvement of the CaSR in the ischemia-induced astroglial reaction. A similar effect of ischemia on receptor expression was described by another report [85]. According to this study, mice subjected to bilateral carotid artery occlusion to induce global cerebral ischemia (GCI) were shown to have an increased CaSR protein expression, especially in CA1 and CA3 of the hippocampus, while concomitantly presenting a reduction in GABABR1 expression in the same regions. These findings were associated with increased neuronal death, and hypothermic treatment prevented both receptor expression changes and cell damage, thus providing evidence for CaSR involvement in neuronal death. Referring to the above cited Chang et al. paper, where an inverse link between the CaSR and GABABR1 expression was described [77], Kim et al. speculated that the reduction of GABABR1 could be a main cause for CaSR overexpression in response to ischemia, and that targeting these receptors could represent an alternative tool for treating ischemic insults [85]. This hypothesis was corroborated by further evidence from genetic and pharmacological approaches. In CaSR-/- mice (with the CaSR KO targeted to the hippocampus and activated three weeks after birth) subjected to transient global cerebral ischemia, a higher survival rate of neurons in the CA1, CA3, and DG regions of hippocampus was observed, compared to the neurons from wild-type animals [86]. In addition, CaSR inhibition by intra-cerebroventricular (ICV) injections of calcilytics effectively protected the hippocampal neurons of wild-type mice from ischemia-induced injury. A pharmacological blockade of the CaSR further preserved neurological abilities in ischemic mice, as assessed by the Morris water maze (MWM) behavioral test, by partly restoring GABABR1 expression [86]. All these findings suggested that inhibiting the CaSR while concurrently stimulating GABABR1 would have represented a key approach to enhance neuroprotection, thus prompting the authors to test the effect of combining calcilytics with the GABABR agonist baclofen in vivo. Interestingly, a co-injection of calcilytics with baclofen had a higher effect in preserving neurons and cell viability from ischemic injury when compared to treatment with a calcilytic alone, therefore confirming the synergism of these compounds to enhance neuroprotection [86].

**Figure 1:**
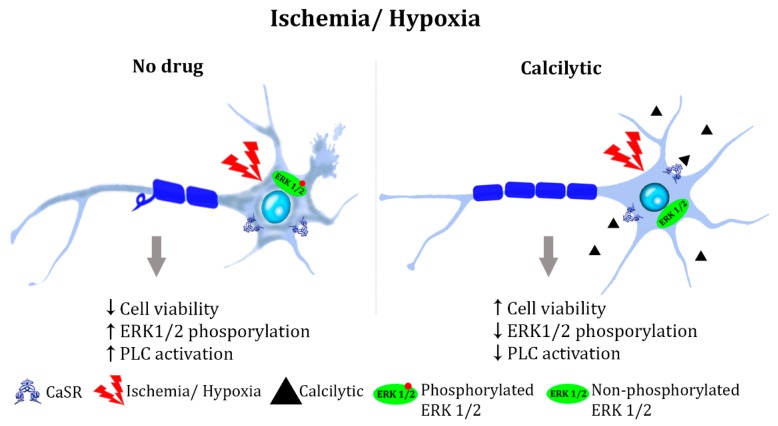
Hypothesized action of a calcilytic in ischemia and hypoxia: Neurons subjected to ischemic or hypoxic stress present reduced cell viability (due to the upregulation of cell death-related proteins such as caspase-3, Bax, and cytochrome C), increased ERK1/2 phosphorylation and phospholipase C (PLC)-activated-Ca^2+^ intracellular mobilization. Such events are likely mediated by the CaSR and concur with cell death (here represented by axonal demyelination and changes in cell morphology). Calcilytic treatment efficiently attenuates mitogen-activated protein kinase (MAPK) and PLC-activated pathways and restores cell viability.

A similar role for the CaSR has been proposed in exacerbating pathological hypoxic processes. Primary cultures isolated from a rat hippocampus and subjected to hypoxia/reoxygenation (H/R) were assessed for cell viability, apoptosis rate, and the expression level of cell death-related proteins such as caspase-3, Bax, and cytochrome C [87]. This resulted in the significant decrease in the hippocampal neuron number and cell viability during H/R, both of which were further reduced when co-treated with the CaSR agonist gadolinium chloride (GdCl_3_). Accordingly, apoptosis rate and the expression of apoptosis-related proteins all significantly increased upon H/R, and as expected, GdCl_3_ further augmented such increase. Conversely, an antagonist of the CaSR, NPS 2390, significantly reversed the H/R-mediated effects. Moreover, the phosphorylation of ERK1/2 was upregulated upon hypoxia/reoxygenation treatment, but it was significantly attenuated by NPS 2390, suggesting that the CaSR is involved in the induction of hippocampus apoptosis during H/R through the phosphorylation of ERK1/2 [87]. In line with this evidence, a recent report by Xue and co-workers showed that NPS 2390 attenuates neuronal apoptosis induced by traumatic brain injury (TBI) [88]. Indeed, a calcilytic reduced the caspase-3 levels, the pro-apoptotic protein Bax, and the release of cytochrome c into the cytosol, which were induced in the TBI-injured rat brains [88]. Similarly, as observed during ischemic injury, hypoxia increased the expression of the CaSR in rat hippocampal neurons and tissue [89]. Moreover, this work showed that intracellular Ca^2+^ mobilization, consequent to the CaSR-mediated PLC activation, was stimulated upon hypoxic stimuli in hippocampal neurons, and the selective blocker of the CaSRCalhex 231inhibited such an increase. In addition, in hippocampal neurons and tissue, hypoxia or GdCl_3_ increased the expression of beta-secretase 1 (BACE1), an enzyme responsible for amyloid precursor protein (APP) cleavage and for the production of the two main amyloid species, Aβ40 and Aβ42. Interestingly, Calhex 231 or Xesto C (a selective inhibitor of IP3 receptor) partly prevented hypoxia-induced BACE1 overexpression. Because these findings supported a link between CaSR activation and amyloid synthesis (a topic that will be discussed in detail in the next section), authors further analyzed the effect of hypoxia on this process. An evaluation of the Aβ40 and Aβ42 contents of rat hippocampal lysates showed that hypoxia or GdCl_3_ raised the content of both amyloid species, and that Calhex 231 or Xesto C partly prevented such increases [89]. To summarize, the mentioned evidences strongly suggest that the CaSR enhances the effects of ischemia and hypoxia, mainly inducing the activation of ERK1/2 and PLC pathways and cell death, therefore indicating calcilytics as potential treatments for these pathological conditions (Figure 1). 

### 4.2. Alzheimer’s Disease

Alzheimer’s disease (AD) is the most common form of dementia, and it accounts for nearly 50 million people worldwide, as recently estimated by the World Health Organization. The biochemical hallmarks of AD are represented by extracellular accumulations of the amyloid peptide Aβ42, known as amyloid plaques [90] and neurofibrillary tangles (NFTs) which are intracellular aggregates of the hyperphosphorylated microtubule-associated protein TAU (MAPT/TAU) [91]. Therefore, the most accredited hypotheses that have been trying to define the key players in AD have pointed at amyloid and TAU, thus proposing them as relevant therapeutic targets [92].

Interestingly, several evidences have supported the CaSR being implicated in the AD pathomechanisms. Early evidence of such involvement showed that Aβ peptides (Aβs) stimulated NCCs in a CaSR-dependent fashion. Indeed, in the cultured hippocampal neurons of wild-type mice, Aβs activated NCCs probably via elevation in cytosolic Ca^2+^, an effect likely mediated by the CaSR, as it was not observed in neurons from mice with constitutive CaSR deletion [93]. Afterwards, the binding between the receptor and soluble Aβ was shown to occur at the plasma membrane of normal adult human astrocytes (NAHAs) by an in situ proximity ligation assay [94]. 

In the last few years, a function of the CaSR in modifying amyloid production and secretion has been supported [95]. As mentioned previously, the pharmacological modulation of the receptor affected amyloid levels of a rat hippocampus [89]. Moreover, an evaluation of Aβ peptides content of NAHAs and human cortical postnatal neurons (HCN-1A) revealed that human neural cells had increased Aβ42 intracellular accumulation and secretion after treatment with exogenous fibrillary Aβ (fAβ25–35) [96]. Remarkably, CaSR pharmacological inhibition with NPS 2143 significantly suppressed the fAβ25–35-mediated surges of endogenous Aβ42 secretion by astrocytes and neurons. Conversely, treatment with the calcimimetic NPS R-568 significantly raised the secreted amount of Aβ42 in NAHAs, thus mimicking effects of the exogenous Aβs [96]. Recent studies have provided new insights regarding the calcilytic-mediated effect on amyloid overproduction. Reports have demonstrated that, in human astrocytes treated with exogenous fibrillary Aβ, NPS 2143 efficiently drove the plasma membrane translocation of both APP and α-secretase ADAM10 and increased the sAPPα secretion over 72 h [97]. According to these findings, calcilytic suppressed the endogenous Aβ42 accrual and secretion driven by fAβ25–35 by restoring the physiological non-amyloidogenic processing of APP and the release of the neurotrophic factor sAPPα. Indeed, Aβ peptides result from amyloidogenic processing, which is initiated by the β-secretase cleavage of the amyloid precursor protein. Alternatively, APP undergoes the most common non-amyloidogenic cleavage, which involves the α-secretase and prevents formation of Aβ species [98]. Thus, authors stated that blocking Aβ/CaSR signaling might counteract amyloid accumulation which occurs during AD [97].

Interestingly, a role of the CaSR in the mechanism underlying phosphorylation of the TAU protein was also proposed. Human astrocytes exposed to fAβ25−35 exhibited an increased activity of glycogen synthase kinase 3 (GSK)-3β responsible for mediating TAU phosphorylation. As a consequence, exogenous fibrillary Aβ significantly increased the production of p-TAU oligomers, which were then released within exosomes. Remarkably, the inhibition of the CaSR with NPS 2143 efficiently abolished the amyloid-mediated effects on GSK-3β and TAU [99], thus giving first evidence of the preventing effect of a calcilytic in TAU phosphorylation. The contribution that the CaSR makes to the pathogenesis of AD was also investigated in vivo on a triple transgenic mouse AD model (3xTg-AD) [100]. Interestingly, semi-quantitative immunohistochemical analyses revealed an augmented expression of the CaSR in the hippocampal CA1 area and in dentate gyrus in the 3xTg-AD mice when compared to non-transgenic control animals. Such an increase was significant at nine months of age, further increased at 12 and 18 months, and it paralleled the accumulation of β-amyloid plaques with age, therefore supporting an interplay between CaSR activation and amyloid accumulation [100]. 

A further contribution to the field was given by a study aimed at investigating the association of CaSR variations in Alzheimer’s disease susceptibility [101]. By using a well-characterized cohort of AD patients and control individuals, authors found a genetic association for the CaSR with AD status only in subjects without an apolipoprotein E (APOE) e4 allele [101], which is reported to be a susceptibility gene for late-onset Alzheimer’s disease [102]. Moreover, using a sensitive luciferase-reporter gene assay, it was demonstrated that exogenous Aβ1-42 as well as APOE activated the CaSR in Cos-1 cells transfected with the receptor [101]. 

**Figure 2:**
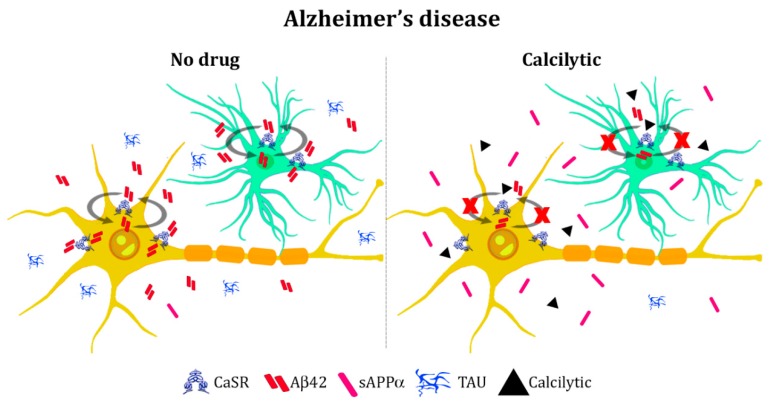
Model of the calcilytic effect in Alzheimer’s disease: Extracellular Aβ42, is over-secreted during Alzheimer’s disease (AD) and binds the CaSR at the plasma membrane of neurons (yellow cell) and astrocytes (green cell). The Aβ42/CaSR-activated signaling mediates a further release of de novo produced Aβ42 and hyperphosphorylated TAU, so feeding a vicious cycle. Moreover, the secretion of sAPPα is dramatically reduced. Treatment with a calcilytic blocks this signaling by reducing Aβ42 and TAU secretion while increasing the release of sAPPα.

Taken together, these studies suggest that interest surrounding the potential involvement of the CaSR in AD has emerged especially during the last decade. To date, studies have reported that the receptor’s modulation affects amyloid and TAU secretion (summarized in Figure 2), but the exact cellular mechanisms need to be understood. Since the postulation of the “amyloid cascade hypothesis” [103], AD research has mainly focused on amyloid as the trigger of neurotoxic events, including TAU phosphorylation and NFTs. Nevertheless, thanks to the formidable increase in knowledge of AD molecular and biochemical mechanisms, researchers have broadened the point of view on the disease and defined it as a multifactorial and complex disorder with several target proteins and cellular processes contributing to its etiology [104]. Ca^2+^ dysfunction has been recognized to play a crucial role in AD. Indeed, the activation of the amyloidogenic pathway produces a remodeling of the neuronal Ca^2+^ signaling, which results in the upregulation of AD and disrupts Ca^2+^ dependent signaling mechanisms, which are responsible for memory formation and learning. On one side, this is due to an increase Ca^2+^ entry through the plasma membrane ion-channel receptors, such as NMDA receptors, activated by extracellular accumulated toxic amyloid species. On the other hand, Ca^2+^ dysfunction is caused by an augmented Ca^2+^ release from internal stores [105,106]. Interestingly, studies reported that clinical mutations in the presenilin genes (PSEN1 and PSEN2), associated with early onset familial AD (EOAD), enhanced Ca^2+^ release from the endoplasmic reticulum (ER) via the inositol 1,4,5-trisphosphate receptors (IP3Rs) and the ryanodine receptors (RyRs) [107], further markedly enhancing calcium signals in AD pathomechanisms. Overall, the remodeled Ca^2+^ signaling system concurs to disrupt the synaptic mechanisms responsible for learning and memory, thus causing neuronal dysfunction and neurodegeneration through the intrinsic activation of calcium-dependent apoptosis [105,108]. Therefore, future studies might unveil a deeper involvement of the CaSR in AD mechanisms.

### 4.3. Tumors of Nervous System

Neuroblastomas are heterogenic tumors of the peripheral nervous system (PNS), originating by the impaired development of neural crest cells. Neuroblastic tumors (such as neuroblastoma, ganglioneuroblastoma, and ganglioneuroma) account for 7–10% of all tumors in children, and 95% of all neuroblastomas occur in individuals under five years of age [109]. Primary lesions are found in the sympathetic ganglia and the adrenal medulla, and therapeutic approaches are based on retinoic acid treatment as a standard therapy to promote tumor cell differentiation [110].

**Figure 3:**
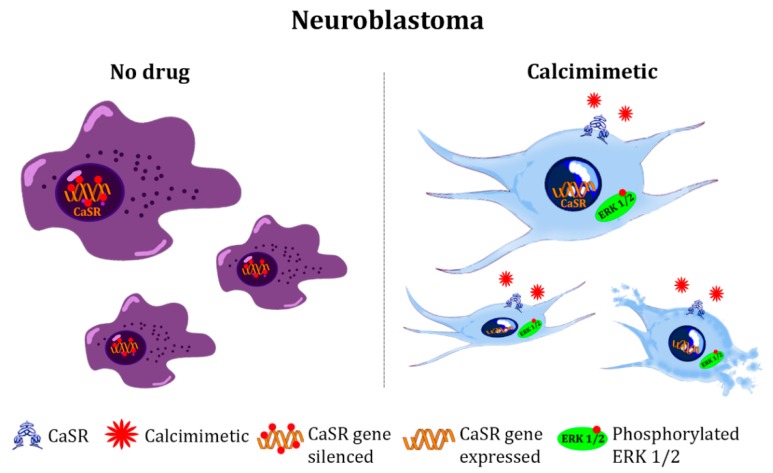
Proposed role of the CaSR in neuroblastoma: The CaSR functions as a tumor-suppressor in neuroblastic tumors. The receptor gene is silenced, while protein expression results to be low or absent in aggressive and high-proliferative neuroblastoma. The CaSR’s restored expression, associated with calcimimetic treatment, promotes tumor differentiation, growth inhibition, and cell death promoted by receptor-activated ERK1/2 phosphorylation.

As previously described in this review, evidence reported the expression of the CaSR in malignancies of the central nervous system (CNS) [57,58]. In those reports, the activation of the CaSR induced the cell proliferation of an astrocytoma cell line. In contrast, several lines of evidence have suggested that the CaSR plays an opposite role in developing nervous system tumors, as the activation of the receptor leads to growth inhibition and induction of differentiation in many neuroblastoma cell lines [111]. An evaluation of CaSR mRNA expression in primary neuroblastic tumors revealed that the receptor expression correlated with good prognostic variables, such as age at diagnosis <1 year, low clinical stage, low clinical risk, and differentiated histology [112]. Interestingly, authors observed that the CaSR, which results in low or non-expression in undifferentiated neuroblastomas, became up-regulated upon inducing differentiation with retinoic acid [112]. Moreover, later studies investigated the mechanisms responsible for silencing the receptor. In particular, promoter 2 hypermethylation and histone modifications of the CaSR gene were found in aggressive neuroblastomas associated with Myc related gene (*MYCN*)-amplification and undifferentiated histology [113]. It is important to note that *MYCN* amplification is the best characterized genetic alteration in human neuroblastomas [114], and it is correlated with a poor prognosis. Interestingly, a treatment with 5-Aza-2′-deoxycytidine (decitabine) and/or trichostatin A (TSA), a demethylating agent and a histone deacetylase inhibitor, respectively, restored CaSR expression in *MYCN*-amplified cell lines [113]. Furthermore, neuroblastoma cell lines stably transfected with the full-length CaSR exhibited a reduced proliferation capacity and resulted in ERK1/2-mediated apoptosis upon activation of the CaSR with high extracellular Ca^2+^ [113], which is in line with the receptor-mediated effect in hypoxic processes. Consistent with these results, a recent study in neuroblastoma cell lines and patient-derived xenografts demonstrated that the calcimimetic cinacalcet inhibited neuroblastoma tumor growth by promoting differentiation, ER stress, and apoptosis [115]. Altogether, these evidences confirmed that the CaSR acts as a tumor-suppressor in neuroblastic tumors by promoting tumor differentiation and cell death through ERK1/2 phosphorylation (Figure 3). A similar function for the receptor has been observed in parathyroid cancer, in which a severe reduction of CaSR expression combined with an increased proliferation has been demonstrated [116]. In this regard, it is noteworthy that parathyroid glands origin from the endoderm of the third and fourth pharyngeal pouches, but they also have substantial contributions from the ectoderm and neural crest [117]. Therefore, neuroblastoma and parathyroids share, at least partially, a common embryological origin from neural crest cells. This might suggest that a similar mechanism for suppressing receptor expression may occur at the level of the neural crest development, which, in turn, may have an impact for driving these tumors. However, no studies have been conducted in this sense. Finally, while several evidences showed that epigenetic modifications are involved in silencing the CaSR in neuroblastoma, the mechanisms responsible for receptor loss in parathyroid tumors have not yet been elucidated.

## 5. Conclusions and Future Perspectives

Evidence accumulated since the first report of CaSR expression in the brain suggests that the receptor is involved in several critical processes underlying the physiological development of neuronal and glial cells, as well as in the proper maintenance and function of the adult nervous system. Furthermore, a critical role of the receptor has been highlighted in neuro-pathological conditions, such as ischemia, Alzheimer’s disease, and in neuroblastoma, and preliminary in vitro and in vivo data suggest that receptor’s modulation might provide novel therapeutic approaches for such disorders. Nevertheless, our understanding of the precise role and signaling of the receptor in the nervous system is largely unknown. Similarly, the exact cellular mechanisms mediated by CaSR modulators that resulted in mitigation of such NS-affecting conditions, need still to be better elucidated. One of the main problems comes from the limits of in vivo models. As mentioned before, the constitutive CaSR KO murine model [43] is characterized by a severe phenotype and early post-natal death, both of which prevents direct in vivo evidence on the adult NS. Certainly, the usage of conditional CaSR deletion models [86] or CaSR KO animals carrying concomitant a genetic ablation of PTH [46] have allowed for the partial overcoming of the limitations of the former model, but further studies are needed to clarify the impact of CaSR absence in the brain. 

An alternative tool for studying the receptor’s relevance for the nervous system might be represented by induced pluripotent stem cell (iPSC) technology [118]. This cutting-edge methodology allows for the reprogramming of adult cells into pluripotent stem cells, which can be further differentiated in functional neurons and glial cells in two- or three-dimensional formats [119]. It has been demonstrated that iPSC systems are particularly helpful in recapitulating the early events of neurogenesis [120] as well modelling human neurodegenerative pathomechanisms in vitro [121]. Moreover, iPSC-derived neuronal cells can also functionally integrate in the nervous circuits when transplanted back into the brain in a mouse model [122]. In addition, genome editing manipulation with the CRISPR/Cas9-system can be successfully applied to the iPSC, thus allowing for the study of the effects of mutated endogenous receptors besides gene knock-out or knock-in mutants in vitro and in vivo. Therefore, by using this technology, the CaSR role and signaling could be ideally elucidated in human-derived neurons or glial cells at different stages of differentiation, and such cultures might also represent more suitable systems for therapeutic aims. Focusing on the therapeutic perspectives of CaSR-targeting drugs, a crucial issue remaining is drug delivery to the CNS. Despite the large number of compounds with therapeutic potential for CNS-diseases, few of these agents are clinically used because of their poor brain barrier penetration. Therefore, pharmacological studies to assess the ability of CaSR modulators to cross the blood-brain barrier and enter the CNS are also needed. However, the growing attention for GPCR’s targeting in nervous system [123], together with progresses of experimental models, makes the advancing of our understanding on the CaSR relevance for the NS a likely possibility in a not too distant future.

## Figures and Tables

**Table 1:** Proposed functions of the CaSR in the cells of developing nervous system.

Cell Types	Model	Role of CaSR	References
**Neurons**	Superior cervical ganglion (SCG) sympathetic neurons	Promotes axonal and dendritic growth and extension through ERK1/2 activation.	[48,49]
	GnRH neuronal cell lines GN11 and GT1-7	Induces neuronal migration and chemotaxis by the secretion of monocyte chemoattractant protein-1, MCP-1; supports the neuronal survival of GnRH neuronal population.	[50]
	Cerebellar granule-cell precursor (GCP) neurons	Stimulates GCPs migration through the activation of MAPK signaling.	[52]
	Neurons differentiated from NSCs of newborn CaSR-/- mice	Serves for neurite growth.	[46]
**Oligodendrocytes**	Oligodendrocytes differentiated from NSCs of newborn CaSR-/- mice	Serves for oligodendrocytes development.	[46]
	Oligodendrocytes differentiated from rat NSCs	Favors oligodendrocyte commitment and lineage progression; stimulates oligodendrocyte proliferation; induces the opening of a Ca-activated K+ Channel.	[54,55]
	20 days post-natal oligodendrocytes from rat brain	Mediates an intracellular calcium mobilization and inositol phosphate accumulation—PLC mediated.	[56]
**Astrocytes**	U373 astrocytoma cell line	Increases cell proliferation; activates a nonselective cation channel (NCC).	[57]
	U87 astrocytoma cell line	Stimulates the opening of an outward K+ channel.	[58]
